# Organizational commitment and its impact on employee performance in the water supply industry: Dataset from Vietnamese state-owned enterprises

**DOI:** 10.1016/j.dib.2024.110029

**Published:** 2024-01-05

**Authors:** Pham Khuong Thao, Nguyen Ngoc-Duy Phuong, Vu Truc Phuc, Nguyen Hong Huan

**Affiliations:** a Institute of Graduate Studies, Hong Bang International University, Ho Chi Minh City, Viet Nam; b Vietnam National University Ho Chi Minh City, Viet Nam; c School of Economics, Finance and Accounting, International University, Viet Nam

**Keywords:** Affective commitment, Continuance commitment, Normative commitment, Job performance, Organizational citizenship behavior

## Abstract

This dataset delves into the intricate dynamics linking organizational commitment (OC) and job performance (JP) within the realm of State-Owned Enterprises (SOEs). Centering on the water supply industry, it seeks to unravel the nuances of organizational citizenship behavior (OCB) in an economy oriented by socialist values. The compilation of this dataset represents a balanced integration of qualitative and quantitative methodologies, incorporating insights from expert interviews alongside data gathered from employee surveys. It encompasses 336 valid responses, which have been meticulously analyzed through Partial Least Squares Structural Equation Modeling (PLS-SEM). This approach facilitates a deeper understanding of the interconnections between OC, OCB, and EP. The dataset is instrumental in highlighting the pivotal role of professional integrity and voluntary dedication within SOEs, underscoring their critical function in representing state interests and effectively serving the public.

Specifications Table

**Table:** 

Subject	Business Management and Decision Sciences
Specific subject area	Human Resource Management, Public Sector Management, Organizational Behaviour.
Data format	Raw
Type of data	Tables, Figures
Data collection	A research instrument was created and verified using qualitative interviews with five experts and conversations with seven people. A 70-employee pilot survey was also done. After then, an initial poll yielded 336 legitimate replies from 400, 64 of which were repetitious. The quantitative data was analyzed using PLS-SEM.
Data source location	City/Town/Region: The research was carried out at Saigon Water Corporation (SAWACO) and all its subsidiaries in Ho Chi Minh City. Country: Vietnam
Data accessibility	Repository name: Mendeley Data Data identification number: 10.17632/tbjdwkzr57.2 Direct URL to data: https://data.mendeley.com/datasets/tbjdwkzr57/2

## 1 Value of the Data

• The dataset provides vital insights into employee commitment in Vietnam's public utilities, focusing on the state-owned water sector.
• A survey was conducted to examine how factors of organizational commitment influence organizational citizenship behavior and job performance in this sector.
• Public sector management improvements can be based on data analysis to improve their dynamics, particularly in state-owned water supply companies.
• Highlighted dataset's potential for comparative studies across industries and cultures.
• Emphasized adaptability of the mixed-methods approach for diverse research areas.
• Detailed the survey design's applicability for cross-industry organizational studies.

## 2 Background

The dataset presents an in-depth exploration of the raw data related to the intricate relationship between organizational commitment (OC) and job performance (JP) in State-Owned Enterprises (SOEs), with a specific focus on the water supply sector. This sector is highlighted for its pivotal role in public service and the socialist market economy. The data provides insights into how various forms of organizational commitment—emotional, continuous, and normative—affect employee behavior and performance in SOEs. A key aspect of the dataset is its analysis of Organizational Citizenship Behavior (OCB) and its impact on employee engagement and contributions beyond formal job requirements, underscoring its importance in public sector service delivery. The dataset is unique in its use of hybrid methods combining both qualitative and quantitative data, offering a comprehensive view of the factors influencing SOE employee commitment, civic behaviors, and their subsequent effects on organizational performance, which are essential for informed policymaking, effective management, and enhancing the efficiency of the public sector.

## 3 Data Description

**Fig. 1:**
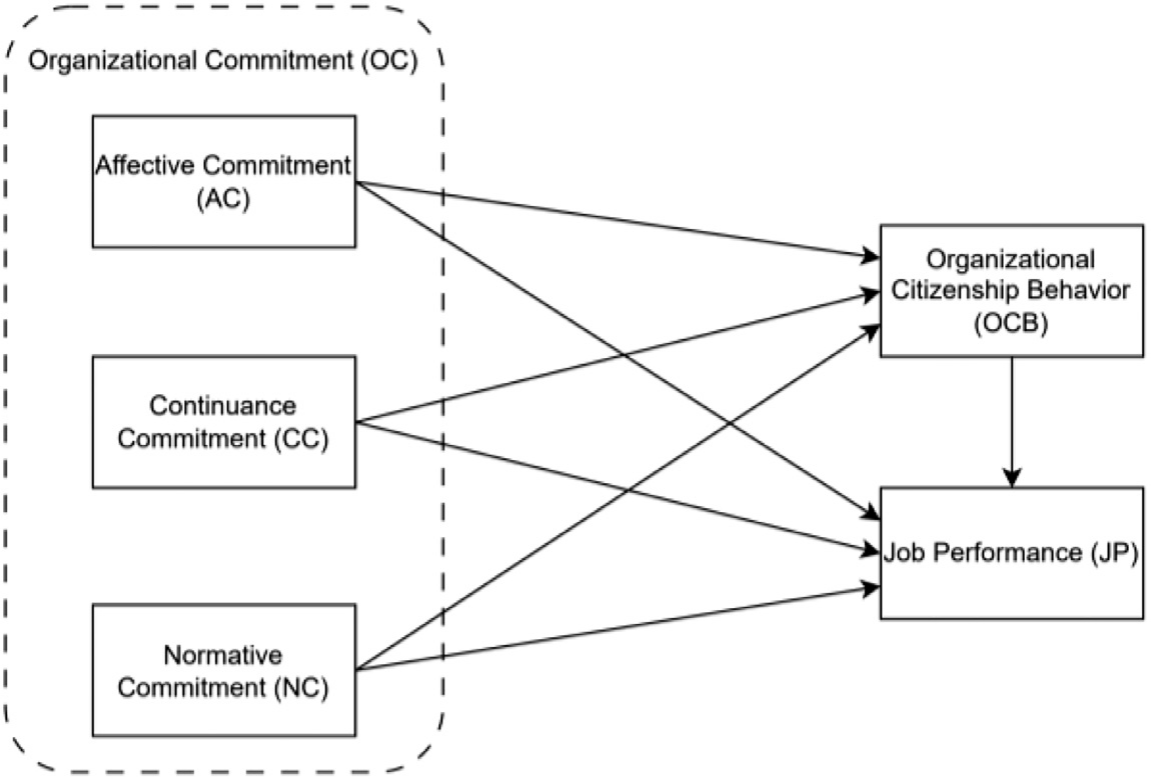
Proposed model.

A survey was conducted between October 2022 and April 2023. It began with a preliminary survey that was given to 70 employees, followed by an extensive survey that elicited 400 responses. Out of these, 336 responses were considered valid for analysis, representing an 84 % response rate. 64 surveys were excluded because of repetitive response patterns. The dataset repository includes two pivotal files, key to elucidating the data's composition and utility. The first file, “Data_OC_OCB_JP.csv”, encapsulates the quantitative data derived from employee surveys. This dataset is meticulously structured, enhancing its suitability for statistical analysis and modeling. It encompasses a diverse array of responses related to OC, OCB, and JP. The second file, “Questionnaire.docx”, is of equal importance; it contains the detailed questionnaire used in the data collection process. The primary survey is structured to encompass three exogenous constructs: Affective Commitment (AC), Continuance Commitment (CC), and Normative Commitment (NC), alongside two endogenous constructs, OCB and JP, as depicted in Fig. 1 based on previous empirical findings of [1,2,[4], [5], [6], [7]]. The multifaceted nature of OC is dissected into three distinct elements: AC, which captures an employee's emotional connection and involvement with their organization, signifying a desire-based commitment; CC, which evaluates the perceived expenses and potential losses entailed in leaving the organization, thus reflecting a commitment rooted in necessity; and NC, which quantifies the felt duty or obligation an employee experiences to stay with their organization, emanating from personal ethical standards or values. These dimensions collectively provide an extensive framework to decipher the complex aspects of an employee's allegiance to their organization. To precisely assess these three dimensions of OC, the survey utilizes specialized scales consisting of six items each for AC, CC, and NC, derived from sources [1], [2], [3]. In addition, the survey incorporates a 10-item scale for OCB, adapted from [4,5], which focuses on voluntary behaviors that contribute positively to organizational efficiency. Complementing this, a 4-item scale for JP, based on [6,7], is integrated to evaluate the effectiveness and proficiency of employees in fulfilling their job responsibilities. These scales together construct a comprehensive and nuanced framework for analyzing the intricate interactions between various factors that influence organizational dynamics and employee performance. The questionnaire items distributed to the participants of this study are listed in Table 1.

**Table 1:** Item wordings for research constructs.

Construct	Item	Description
Affective Commitment (AC)	AC1	I am happy to dedicate the rest of my career to SAWACO.
	AC2	I feel as if the problems of SAWACO are my own.
	AC3	I felt I “belonged” to SAWACO.
	AC4	I feel “emotionally attached” to SAWACO.
	AC5	I feel “part of the family” at SAWACO.
	AC6	SAWACO means a lot to me personally.
Continuance Commitment (CC)	CC1	Currently, staying with SAWACO is a necessity and a desire.
	CC2	It would be challenging to leave SAWACO right now, even if I wanted to.
	CC3	My life will be significantly disturbed if I decide to leave SAWACO now.
	CC4	I have too few other options to consider if I leave SAWACO.
	CC5	I will devote myself to SAWACO, and it is difficult for me to choose to work elsewhere.
	CC6	One of the few negative consequences of leaving SAWACO is that it is difficult to find alternative jobs available.
Normative Commitment (NC)	NC1	I felt obliged to stay and work with SAWACO.
	NC2	Even if it was to my advantage, I felt it was wrong to leave SAWACO now.
	NC3	I would feel guilty if I left SAWACO now.
	NC4	SAWACO deserves my loyalty.
	NC5	I will not leave SAWACO right now because I am responsible to the people in it.
	NC6	I owe SAWACO a lot.
Organizational Citizenship Behavior (OCB)	OCB1	I spent time guiding, mentoring, or counseling a colleague.
	OCB2	I aided my co-workers in acquiring new abilities or imparted my job knowledge.
	OCB3	I supported new employees in familiarizing themselves with their roles.
	OCB4	I provided a sympathetic ear to colleagues facing work challenges.
	OCB5	I proposed ideas for enhancing work processes.
	OCB6	I assisted a colleague who was overwhelmed with their workload.
	OCB7	I willingly took on additional work tasks.
	OCB8	I worked during weekends or on my days off to ensure the completion of projects or tasks.
Job Performance (JP)	JP1	Quality of my performance.
	JP2	My productivity on the job
	JP3	My colleagues' performance compared to me doing the same type of work as mine
	JP4	My performance compared to my colleagues doing the same type of work as theirs

Responses in the survey were measured using a 7-point Likert-type scale, ranging from 1 (strongly disagree) to 7 (strongly agree). The mean values, ranging from 4.71 to 5.50. The standard deviation and variance values indicate the spread and dispersion of responses. Skewness and kurtosis values provide insights into the distribution, respectively. This detailed statistical presentation is crucial for understanding the nuances in the response patterns and assessing the reliability of the constructs measured in the dataset. Table 2 presents essential statistical measures of the data, including Mean, Standard Deviation, Skewness, and Kurtosis.

**Table 2:** Descriptive statistics of the constructs' items.

Item	Mean	Std. Deviation	Variance	Skewness		Kurtosis	
					Std. Error		Std. Error
AC1	4.97	1.252	1.566	−0.464	0.133	−0.131	0.265
AC2	5.01	1.261	1.591	−0.672	0.133	0.211	0.265
AC3	4.96	1.276	1.628	−0.624	0.133	0.220	0.265
AC4	4.81	1.384	1.916	−0.456	0.133	−0.292	0.265
AC5	4.90	1.353	1.830	−0.547	0.133	−0.159	0.265
AC6	5.03	1.394	1.942	−0.618	0.133	0.124	0.265
CC1	5.01	1.401	1.964	−0.652	0.133	−0.002	0.265
CC2	5.14	1.337	1.788	−0.550	0.133	−0.072	0.265
CC3	5.09	1.418	2.010	−0.829	0.133	0.557	0.265
CC4	5.18	1.282	1.644	−0.828	0.133	0.675	0.265
CC5	5.14	1.354	1.834	−0.836	0.133	0.702	0.265
CC6	5.28	1.300	1.690	−0.783	0.133	0.581	0.265
NC1	4.98	1.269	1.612	−0.495	0.133	0.131	0.265
NC2	4.84	1.240	1.538	−0.409	0.133	0.010	0.265
NC3	5.12	1.222	1.493	−0.680	0.133	0.708	0.265
NC4	4.79	1.419	2.014	−0.509	0.133	−0.214	0.265
NC5	4.96	1.228	1.509	−0.359	0.133	−0.446	0.265
NC6	4.92	1.318	1.737	−0.636	0.133	0.083	0.265
OCB1	5.14	1.242	1.544	−0.734	0.133	0.672	0.265
OCB2	5.50	1.224	1.498	−1.132	0.133	1.642	0.265
OCB3	5.17	1.196	1.431	−0.784	0.133	0.929	0.265
OCB4	5.16	1.238	1.532	−0.859	0.133	1.010	0.265
OCB5	4.75	1.483	2.198	−0.603	0.133	−0.087	0.265
OCB6	4.71	1.494	2.232	−0.637	0.133	−0.059	0.265
OCB7	4.91	1.361	1.852	−0.758	0.133	0.303	0.265
OCB8	4.94	1.382	1.910	−0.608	0.133	−0.026	0.265
EP1	5.21	1.227	1.506	−0.864	0.133	1.147	0.265
EP2	5.01	1.302	1.695	−0.740	0.133	0.519	0.265
EP3	4.83	1.374	1.889	−0.620	0.133	0.060	0.265
EP4	4.74	1.402	1.966	−0.656	0.133	0.113	0.265

Table 3 presents statistical measures applied to key constructs such as AC, CC, NC, OCB, and JP. It includes Factor Loadings, which range from 0.707 to 0.935 and indicate the correlation strength between each item and its construct. Higher values signify greater reliability. The Variance Inflation Factor (VIF) assesses multicollinearity and shows values between 1.320 and 4.459, which are within acceptable limits. The table also shows Cronbach's Alpha (CA) and Composite Reliability (CR) values, which are both measures of internal consistency. The figures range from 0.737 to 0.929, indicating strong reliability [8,9]. Average Variance Extracted (AVE) values highlight the variance captured by the construct as opposed to measurement error. They range from 0.639 to 0.825, surpassing the acceptable threshold of 0.5 [9].

**Table 3:** Result of reliability and convergent validity.

	Factor Loadings	VIF	CA	CR	AVE
AC1	0.894	3.199	0.908	0.91	0.783
AC4	0.905	3.301			
AC5	0.895	2.962			
AC6	0.844	2.345			
CC1	0.891	3.011	0.929	0.929	0.825
CC2	0.935	4.459			
CC4	0.899	3.185			
CC6	0.908	3.379			
JP1	0.847	1.647	0.737	0.755	0.656
JP2	0.728	1.320			
JP4	0.849	1.568			
NC1	0.829	3.241	0.922	0.923	0.72
NC2	0.852	3.654			
NC3	0.833	2.647			
NC4	0.852	3.296			
NC5	0.890	3.600			
NC6	0.836	2.526			
OCB1	0.866	3.019	0.905	0.915	0.639
OCB2	0.834	2.528			
OCB3	0.762	2.060			
OCB4	0.816	2.512			
OCB5	0.707	1.921			
OCB7	0.833	3.187			
OCB8	0.767	2.557			

**Fig. 2:**
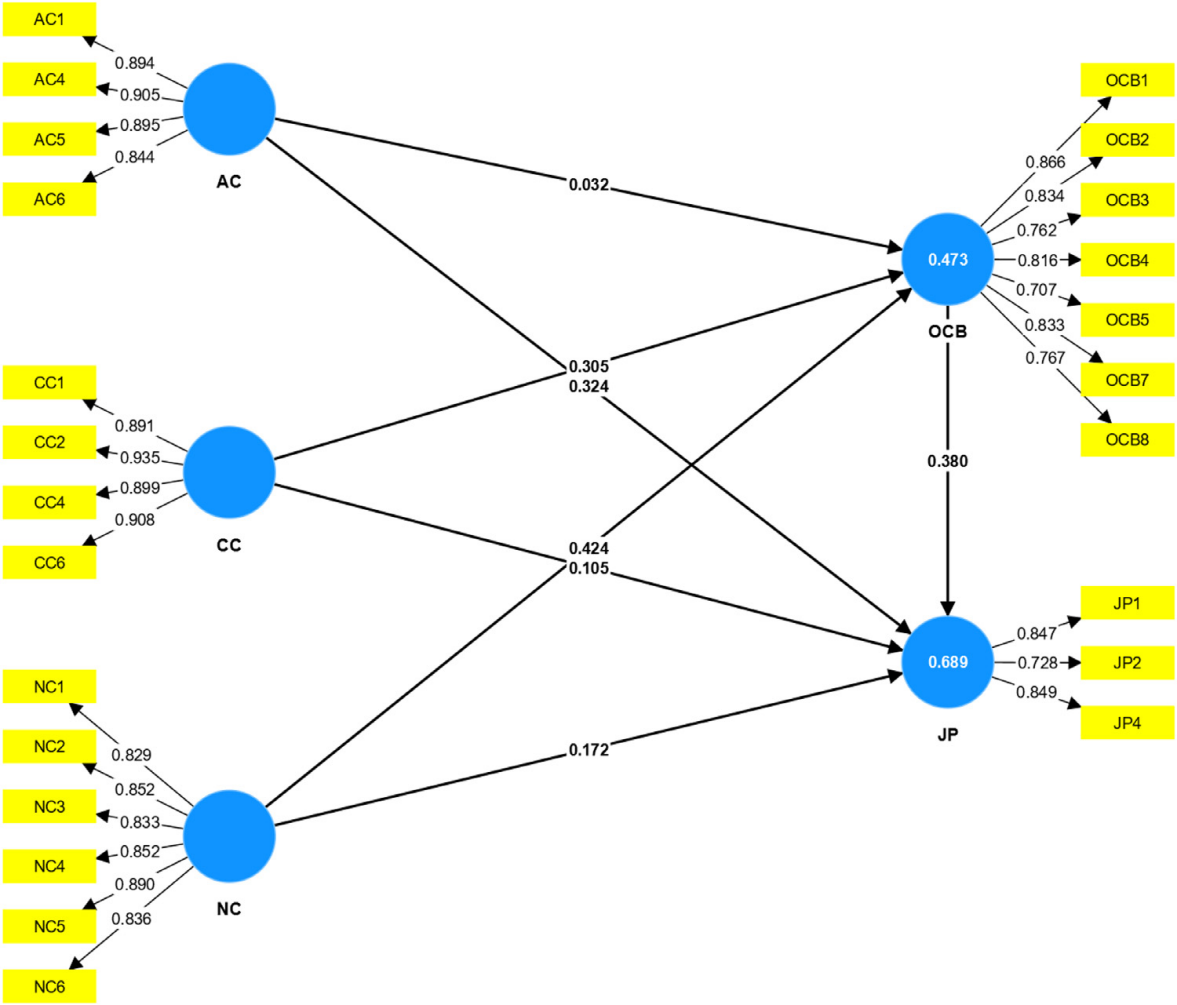
The PLS-SEM result.

Table 4 focuses on the Fornell-Larcker Criterion [10] and examines discriminant validity by contrasting the square root of AVE values (diagonal) against the correlations between constructs (off-diagonal). The diagonal values, between 0.799 and 0.908, exceed the off-diagonal values, ensuring adequate discriminant validity. The structural representation of the measurement model of the dataset is illustrated in Fig. 2.

**Table 4:** Fornell-Larcker criterion.

	AC	CC	JP	NC	OCB
AC	**0.885**				
CC	0.608	**0.908**			
JP	0.700	0.643	**0.810**		
NC	0.690	0.655	0.709	**0.849**	
OCB	0.510	0.602	0.719	0.646	**0.799**

Table 5 provides VIF values (inner model), which are a measure of how much the variance of an estimated regression coefficient increases due to multicollinearity. In the raw data context, VIF values give an initial indication of the degree of correlation between independent variables. In this dataset, the VIF values range from 1.899 to 2.636. These figures suggest that in the raw survey data, each independent variable does not overly inflate the variance of the others, indicating a low to moderate level of multicollinearity which is within acceptable limits in most regression analyses. It suggests that the constructs are relatively independent in the raw data.

**Table 5:** Collinearity statistic (VIF) – inner model.

	VIF
AC -> JP	2.077
AC -> OCB	2.075
CC -> JP	2.084
CC -> OCB	1.907
NC -> JP	2.636
NC -> OCB	2.295
OCB -> JP	1.899

Table 6 presents the path coefficients, including direct and indirect effects, which show the strength and direction of the relationship between constructs. Direct path coefficients such as AC -> JP (0.324) suggest a strong positive relationship in the raw data between affective commitment and job performance. Indirect paths like AC -> OCB -> JP (0.012) represent the mediated relationship, where the effect of AC on JP through OCB is very small. These coefficients are derived from the raw data and illustrate how changes in one variable are associated with changes in another, in the context of the survey responses.

**Table 6:** Path coefficients.

	Path coefficients
AC -> JP	0.324
AC -> OCB	0.032
CC -> JP	0.105
CC -> OCB	0.305
NC -> JP	0.172
NC -> OCB	0.424
OCB -> JP	0.38
NC -> OCB -> JP	0.161
AC -> OCB -> JP	0.012
CC -> OCB -> JP	0.116

Table 7 contains f-square values which indicate the size of the effect an independent variable has on a dependent variable. For example, an f-square value of 0.163 for AC -> JP in the raw data suggests a medium effect size, meaning that affective commitment has a moderately strong association with job performance in the raw survey responses. Meanwhile, a value like 0.001 for AC -> OCB implies that the effect of affective commitment on organizational citizenship behavior is almost negligible in the raw data. These effect size measures provide insight into the relative impact of the independent variables on the dependent variables directly from the collected data.

**Table 7:** f-square.

	f-square
AC -> JP	0.163
AC -> OCB	0.001
CC -> JP	0.017
CC -> OCB	0.092
NC -> JP	0.036
NC -> OCB	0.148
OCB -> JP	0.244

The structural representation of the measurement model of the dataset is illustrated in Fig. 2.

## 4 Experimental Design, Materials and Methods

This research adopted a mixed-methods approach to gain a comprehensive understanding of organizational commitment within the water supply industry by seamlessly integrating qualitative and quantitative methodologies. First, a comprehensive review of relevant literature guided the development of key constructs, and questions were crafted to capture these, ensuring clarity and relevance. The questionnaire underwent a rigorous validation process, including expert reviews and pilot testing with a group representative of the study population, leading to refinements based on the feedback received. In the initial qualitative phase of data collection, a series of in-depth one-on-one discussions with a select group of five experts, including two seasoned professionals from the water supply sector and three scientists, were conducted. These discussions were essential for clarifying and refining the constructs related to OC, OCB, JP. The selection of these experts was strategically done, adhering to the principle of information saturation, where the number of interviews was determined based on the emergence of no new information. The insights obtained from these interactions were pivotal in tailoring the measurement scales to suit the specific context of the Vietnamese water supply industry, particularly focusing on Ho Chi Minh City. This process involved a meticulous adaptation and translation of existing scales from prior studies, ensuring the precision and comprehensibility of the questionnaires. These refined scales subsequently formed the basis of a preliminary questionnaire, which was further evaluated and improved through a focused group discussion with seven key personnel from the Saigon Water Corporation. This phase was crucial in providing a thorough understanding of the context and played an instrumental role in the validation and refinement of the quantitative survey instruments. Through these qualitative interactions, the questionnaire items were meticulously modified and clarified to enhance their relevance and comprehension in the specific context of the water supply industry. After the qualitative phase, data collection was conducted via in-person interviews, email, and an online questionnaire. The questionnaire, which included a declaration on data ethics and confidentiality, had two primary sections. The first part gathered gender, marital status, age, education, and position. The key research variables in the second segment were assessed using a 7-point Likert scale with 30 items Table 8 provides details on the sample characteristics, including frequency and percentage tests.

**Table 8:** Frequencies statistics for screening and demographic questions.

Characteristics	*N* = 336	Percentage (%)
*Gender*		
Male	238	70.80
Female	98	29.20
		
*Marital status*		
Single	18	5.36
Married	318	94.64
		
*Age*		
18–24	6	1.80
25–35	99	29.50
36–45	125	37.20
46–55	64	19.00
>55	42	12.50
		
*Education*		
High School	86	25.60
College	52	15.50
University	178	53.00
Master	17	5.10
Doctorate	3	0.90
		
*Position*		
Board of Directors	13	3.90
Chief/Deputy Chief of Staff	42	12.50
Manager/Leader	127	37.80
Employee	56	16.70
Worker	98	29.20

A rigorous and diverse statistical procedure was used throughout data processing to assure survey construct robustness and validity. To assess item-latent variable association strength and relevance, we investigated construct factor loadings. Verifying each item's build representation was critical. For each build, we calculated CA and CR to assess internal consistency and assure dependability. We also added composite reliability for each construct to ensure the measurement scales' reliability and internal consistency. The AVE calculation confirmed that a large percentage of each construct's variation was attributable to the latent variable, not measurement error, thus confirming convergent validity. To avoid multicollinearity, we determined the VIF to ensure that the constructs were distinct and not highly associated. We assessed discriminant validity using the Fornell-Larcker and HTMT criteria. The Fornell-Larcker criteria compare the square root of the AVE of each construct-to-construct correlation to ensure that each construct is more strongly linked with its indicators than others. Furthermore, we computed the HTMT ratio [11], a more current and stricter metric, to demonstrate the constructs' uniqueness and absence of overlap. This thorough data analysis enabled us to rigorously assess the theoretical model and laid the foundation for SEM, which accurately examined the expected construct correlations.

## Limitations

This study, focusing on organizational commitment and job performance within Vietnamese State-Owned Enterprises in the water supply industry, faces several limitations. The research's scope, confined to a specific sector and cultural context, limits the generalizability of the findings to other industries or geographic regions. The methodological reliance on self-reported data from a specific number of employees may introduce response biases, potentially skewing the results. Additionally, the study's cross-sectional design precludes drawing definitive conclusions about cause-and-effect relationships. Furthermore, the exclusion of 64 responses due to repetitive patterns raises questions about the representativeness of the survey sample. Lastly, while the mixed-methods approach enriches the study, the integration of qualitative and quantitative data may present challenges in ensuring methodological consistency and interpretive coherence.

## Ethics Statement

All authors involved in the research followed ethical standards. Prior to their participation, all participants were fully informed about the research's goals and nature. Informed consent was obtained from all participants, who cannot be contacted now. The study did not require approval from the Institutional Review Board (IRB). All data collected during the study was anonymized to protect participant confidentiality. Personal identifiers were removed, and unique codes were used to replace any potentially identifying information. This process ensured the privacy and confidentiality of all respondents, in accordance with ethical research practices.

## CRediT authorship contribution statement

**Pham Khuong Thao:** Conceptualization, Methodology, Software, Data curation, Investigation. **Nguyen Ngoc-Duy Phuong:** Conceptualization, Formal analysis, Writing – review & editing, Supervision. **Vu Truc Phuc:** Conceptualization, Supervision. **Nguyen Hong Huan:** Conceptualization, Software, Validation, Formal analysis, Visualization, Writing – review & editing.

## Data Availability

Dataset Examining the Relationship Between Organizational Commitment and Job Performance in Vietnam's Water Supply Sector (Original data) (Mendeley Data)
